# Comparison of apical microleakage using Ni-Ti with stainless steel finger spreaders

**Published:** 2009-10-10

**Authors:** Shahriar Shahi, Sahar Shakouie, Saeed Rahimi, Hamid Reza Yavari, Narmin Mohammadi, Majid Abdolrahimi

**Affiliations:** 1 *Department of Endodontics, Dental School, Tabriz University of Medical Sciences, Tabriz, Iran*; 2 *Iranian Center for Endodontic Research, Tehran, Iran*

**Keywords:** Microleakage, NiTi, Spreader, Stainless Steel

## Abstract

**INTRODUCTION:** The purpose of this *in vitro* study was to compare apical microleakage after obturation with Nickel-Titanium (NiTi) compared to Stainless Steel (SS) finger spreaders.

**MATERIALS AND METHODS:** Eighty straight single-rooted human teeth were instrumented using step-back technique. The specimens were randomly divided into four groups. The two experimental groups (n=30) and the negative control group (n=10) were obturated by lateral condensation technique with Ariadent gutta-percha and AH26 sealer. The roots in the positive control group (n=10) were instrumented but not obturated. In one group, SS and in another group NiTi spreaders were used. Microleakage evaluation was conducted using dye penetration method t-test was used for statistical analysis.

**RESULTS:** The results showed statistically significant differences between NiTi and SS groups (P=0.022), with the greatest dye penetration in SS group and the least in NiTi group.

**CONCLUSION:** According to the results of the present study using NiTi spreader decrease apical microleakage in endodontically treated teeth. [Iranian Endodontic Journal 2009;4(4):149-51]

## INTRODUCTION

The quality of obturation is of utmost importance for successful endodontic therapy by preventing leakage of microorganisms and their by-products into the periradicular tissues (1). A commonly used obturation technique is cold lateral compaction of gutta-percha (GP) with sealer (1-3). Studies have demonstrated that the best apical seal is achieved when the spreader is placed close to working length (4-6). Stainless Steel (SS) spreaders may fail to reach this depth because of resistance occurring as a result of binding against canal walls (e.g. curved canals) (7). This inflexibility might lead to an insufficient apical seal or possibly vertical root fractures (8,9).

It has been reported that deeper spreader penetration and less required force with Nickel-Titanium (NiTi) spreaders compared with SS spreaders. They showed that in teeth with curved canals, NiTi spreaders penetrate to a greater extent than SS spreaders when firm resistance to spreader advancement was used to measure spreader penetration in extracted teeth (7). Therefore, using NiTi spreaders may minimize the potential for microleakage during lateral compaction. The purpose of this study was to compare apical microleakage of NiTi and SS finger spreaders packed canals in extracted teeth.

## MATERIALS AND METHODS

Eighty single root canals with canal lengths of at least 10 mm (with less than 5 degrees curvature) were used for this *in vitro* study. Samples were chosen from a storage container of sodium hypochlorite (NaOCl) 2.6%. Before preparation, the teeth were treated with NaOCl 5.25% for 60 minutes to remove the periodontal ligaments and were stored in thymol 0.2%. Straight-line access cavities were obtained in all the specimens; then a K-file size #10 (Maillefer, Ballaigues, Switzerland) was placed in each canal until the file tip was flush with the external surface of the tooth. One millimeter was subtracted from this length to establish the working length. The canals were cleaned and shaped using the step-back method, and a K-Flexofile size #35 (Maillefer, Ballaigues, Switzerland) was chosen as the master apical file (MAF). The canals were then flared up to file size #60 (K-Flexofile, Maillefer, Ballaigues, Switzerland). Irrigation with 2 mL NaOCl 2.5% and recapitulation with K-Flexofile size #35 to working length was accomplished after each filing procedure. Then, teeth were randomly divided into two experimental groups. In one group obturation was carried out using SS finger spreader and other using Ni-Ti finger spreader. In each experimental group, 5 teeth were considered as positive controls and 5 as negative controls.

**Table 1:** Comparison of microleakage between experimental groups using t-test

Groups	n	Mean±SD (mm)	Standard Error	t	df	p
**Stainless steel**	30	3.3267±1.5359	0.02804	2.33	58	0.022
**NiTi**	30	2.4333±1.4095	0.2573			

Root canals were obturated using lateral compaction method with a #35 Diadent (Diamond dental, Chongiu, Korea) master cone and accessory cones (Ariadent, Tehran, Iran) with AH26 sealer (Dentsply, Konstanz, Germany). Master cone tug-back was confirmed by the resistance of the cone to removal from the canal, and a radiograph confirmed the working length. The master cones were coated with sealer and placed in the canals. A SS finger spreader size #25 in one group, and a NiTi finger spreader size #25 in another group were inserted into the canals for 5 seconds on each penetration, and nonstandard gutta-percha (MF) was condensed against the canal wall using the lateral compaction technique. After removal of the spreader from the canal, accessory GP cones were coated with sealer and inserted in the space left by the spreader removal. On average, 5-7 accessory cones were placed into each canal. Proximal view radiographs were used for evaluation of the obturation quality. Positive control included root canals which were instrumented but not filled and the negative control contained root canals which were instrumented and obturated, but the entire surfaces of the specimen in this group were covered with fingernail varnish and sticky wax. All samples were decoronated at the cement-enamel junction (CEJ). The specimens were stored at 100% humidity at 37^◦^C for 7 days. Then the root surfaces of all the specimens except for apical foramina were coated with fingernail varnish and sticky wax. The latter procedure was not carried out for negative controls. The apical foramina of the teeth in the positive controls were allowed to remain patent to determine if leakage would occur throughout the length of the canal. The teeth in the negative control group were coated with fingernail varnish and sticky wax. All the specimens were submerged in a 2.5% solution of methylene blue for 72 h. Vertical grooves were carved on the buccal and palatal walls of all the specimens by diamond disk (D & Z, Munchen, Germany) and the teeth were split longitudinally. GP was removed and a stereomicroscope was used to assess microleakage. The extent of microleakage was determined by linear measurement of dye penetration and 0.1 mm accuracy. The extent of dye penetration into the specimens was measured separately by two individuals at two different times, and the mean value of the recorded measurements was chosen as the extent of dye penetration into each specimens. The data were analyzed using SPSS and two experimental groups were compared using t-test.

## RESULTS

No dye penetration was observed in the negative control group, and dye penetration into the entire canal length was observed in the positive control group. The result of the study indicated that mean values of dye penetration were 3.32±1.53 mm in SS group and 2.43±1.40 mm in NiTi group. T-test showed that the differences between the groups were statistically significant (P=0.022), with the greatest and least dye penetration in SS group and NiTi group, respectively (Table 1).

## DISCUSSION

Our statistical analysis demonstrated that NiTi spreader in the lateral condensation technique leads to less microleakage and a better seal compared to SS spreaders. Placing the spreader 1-2 mm above the apical foramen for proper canal obturation has been demonstrated (5). Shahi *et al.* showed that spreader penetration up to the working length results in a better seal compared to penetration 1 mm less than that (4). Wilson demonstrated deeper NiTi spreader penetration compared to SS spreader with 0.04 tapered GP points and 1.5 kg compaction force during canal obturation (10). Using heavy apically-directed force with spreaders to achieve working length is one of the important reasons for vertical root fracture. Less force is required with NiTi spreaders to penetrate to the apical 1 mm of the root compared to stainless spreaders. This is considered a factor to decrease the incidence of vertical root fractures; also a better seal is achieved by this deep penetration. In the present study the spreader penetrated to working length in both groups. Since NiTi spreader penetration is greater than that in SS spreaders, the space produced permits deeper penetration of accessory GP points, which can be considered a contributing factor in creating a better seal (11). A study carried out by Sobhi demonstrated a deeper NiTi spreader penetration compared to stainless spreader at a given force without producing voids in the filling (11), which is consistent with the results of the present study.

Dye penetration technique was used in the present study to evaluate apical microleakage. In this technique, the reasons for microleakage cannot be determined. The dye used was methylene blue which is composed of sub-microbe-sized particles, increasing the accuracy of the evaluation. If such particles cannot penetrate into the space between the canal walls and GP, large particles such as bacteria and their endotoxins will not penetrate, either (12). In this study, difference in dye penetration can be attributed to the type of the spreaders used. Other variables (*e.g.* sealer, GP, compaction techniques and force for obturation, storage conditions, and the methods used for apical microleakage evaluation were similar.

## CONCLUSION

NiTi spreader decreases microleakage of endodontically treated teeth; however, further *in vivo* evaluations are needed to assess the effect of NiTi spreaders on apical seal and healing of periapical lesions. 
